# Methionine Sulfoxide Reductase B Regulates the Activity of Ascorbate Peroxidase of Banana Fruit

**DOI:** 10.3390/antiox10020310

**Published:** 2021-02-18

**Authors:** Lu Xiao, Guoxiang Jiang, Huiling Yan, Hongmei Lai, Xinguo Su, Yueming Jiang, Xuewu Duan

**Affiliations:** 1 Key Laboratory of Plant Resource Conservation and Sustainable Utilization, South China Botanical Garden, Chinese Academy of Sciences, Guangzhou 510650, China; xiaolu@scbg.ac.cn (L.X.); gxjiang@scbg.ac.cn (G.J.); hlingyan@scbg.ac.cn (H.Y.); laihongmei@scbg.ac.cn (H.L.); ymjiang@scbg.ac.cn (Y.J.); 2 Center of Economic Botany, Core Botanical Gardens, Chinese Academy of Sciences, Guangzhou 510650, China; 3 College of Advanced Agricultural Sciences, University of Chinese Academy of Sciences, Beijing 100049, China; 4 Guangdong AIB Polytechnic, Guangzhou 510507, China; suxg@gdyzy.edu.cn

**Keywords:** ascorbate peroxidase, reactive oxygen species, methionine sulfoxide reductase, sulfoxidation, posttranslational modification

## Abstract

Ascorbate peroxidase (APX) is a key antioxidant enzyme that is involved in diverse developmental and physiological process and stress responses by scavenging H_2_O_2_ in plants. APX itself is also subjected to multiple posttranslational modifications (PTMs). However, redox-mediated PTM of APX in plants remains poorly understood. Here, we identified and confirmed that MaAPX1 interacts with methionine sulfoxide reductase B2 (MsrB2) in bananas. Ectopic overexpression of *MaAPX1* delays the detached leaf senescence induced by darkness in Arabidopsis. Sulfoxidation of MaAPX1, i.e., methionine oxidation, leads to loss of the activity, which is repaired partially by MaMsrB2. Moreover, mimicking sulfoxidation by mutating Met36 to Gln also decreases its activity in vitro and in vivo, whereas substitution of Met36 with Val36 to mimic the blocking of sulfoxidation has little effect on APX activity. Spectral analysis showed that mimicking sulfoxidation of Met36 hinders the formation of compound I, the first intermediate between APX and H_2_O_2_. Our findings demonstrate that the redox state of methionine in MaAPX1 is critical to its activity, and MaMsrB2 can regulate the redox state and activity of MaAPX1. Our results revealed a novel post-translational redox modification of APX.

## 1. Introduction

Reactive oxygen species (ROS) are formed as natural byproducts during cellular metabolism and play important roles in cell signaling and homeostasis [1,2]. However, under stress conditions or during senescence, excess ROS accumulation can cause oxidative damage to macromolecules such as proteins, DNA, and lipids, which, in turn, results in loss of structure and function or even potential cell death [1,2,3,4]. To counteract oxidative stress, organisms have evolved complex protective systems, such as antioxidant systems and macromolecule repair systems [5].

The enzymatic antioxidant system constitutes the most critical protective mechanism in plants, including ascorbate peroxidase (APX), catalase, superoxide dismutase, glutathione peroxidase, and peroxidase [1,6,7]. In addition, some low molecular mass antioxidants (ascorbate, glutathione, tocopherols, and phenolic compounds) are involved in scavenging excess ROS in the plant [8]. Among the antioxidant enzymes, APX plays a crucial role in scavenging H_2_O_2_ by catalyzing the conversion of H_2_O_2_ to H_2_O and O_2_, using ascorbic acid as the electron donor [9]. APX has a higher affinity than does catalase for H_2_O_2_ and contributes maximally to H_2_O_2_ detoxification in chloroplasts, cytosol, mitochondria, and peroxisomes, as well as in the apoplastic space [10]. APXs are known to be involved in the physiological and developmental response, such as seed germination [11], leaf senescence [12], and programmed cell death [13]. APX also participates in environmental stresses in plants, including drought [14], salt [15], chilling [16,17], photo-oxidative stress [18], and high temperature [19]. 

Proteins are one of the main targets of oxidative damage. Oxidative stress can cause irreversible or reversible modification of proteins. Irreversible oxidation, such as carbonylation, generally leads to impairment of protein function [20]. Reversible oxidation, particularly at methionine and cysteine residues, can be repaired by the Msr and thioredoxin (Trx) systems, respectively. Met oxidation forms two diastereomers of methionine sulfoxide (Met-*S*-O and Met-*R*-O), which can be reduced by the methionine sulfoxide reductases, MsrA and MsrB, respectively. Previous studies on Msr have mainly focused on its role in resistance to oxidative stress in organisms [21,22,23,24], which is related to repair of oxidized proteins, such as GroEL [25], Fth [26], hERG [27], apolipoprotein A-I [28], CaMKII [29], TRPM6 channel [30], HypT [31], actin [32], CaM [33,34], GrpEL1/Mge1 [35], heme oxygenase [36], and high-density lipoprotein [37]. Growing evidence suggests that Msr may be implicated in the regulation of protein function by modifying the sulfoxidation of proteins in a similar manner to that of other protein modification, such as phosphorylation [38,39,40,41]. However, only a few potential targets of Msr in relation to sulfoxidation modification have been investigated in higher plants [23,34,42]. 

Seed-bearing fruits in flowering plants provide humans with an important food source. Senescence is a vital stage of fruit life and directly decreases fruit quality and reduces resistance to pathogens, which usually cause enormous economic losses. Most fruits after harvest undergo vigorous aerobic respiration, accompanied by the accumulation of ROS. Recent discoveries have revealed that fruit senescence is considered to be related to ROS accumulation and oxidative damage of protein [43]. Therefore, elimination of ROS and repair of oxidized proteins are crucial for the maintenance of the function of the proteins and regulation of senescence. 

Banana (*Musa acuminate* L.) is one of the most economically important fruits, undergoing rapid ripening and senescence once harvested. Our preliminary study found that one APX (GSMUA_Achr5P07280_001), designated as MaAPX1, might be a target of MaMsrB2 by Co-IP and mass spectrum techniques in banana fruit [44]. Here, we evaluated the possible role of MaAPX1 in regulating senescence by heterologous expression of MaAPX1 in *Arabidopsis thaliana*. We then validated that MaAPX1 was a substrate of MaMsrB2 and reduction of oxidized Met in MaAPX1 by MaMsrB2 reversibly switched on the MaAPX1 activity. In addition, mimicking oxidation of Met36 in MaAPX1 further confirmed that the redox state of Met36 can influence MaAPX1 activity. Furthermore, the mechanism underlying MaMsrB2-mediated regulation of MaAPX1 activity was further investigated by spectroscopic analysis. Our results revealed a novel mechanism of post-translational redox modification of APX.

## 2. Materials and Methods 

### 2.1. Plant Materials and Treatments

Green mature banana (*Musa acuminata* L.) fruit (approximately 110 days after anthesis) were harvested from an orchard in Guangzhou, Guangdong province, China. Fruit with uniform shape, color, and size were selected and grouped into two lots. One group was stored under 60% oxygen concentration at 25 °C and 85-90% relative humidity. The other group was stored under normal air conditions as the control. During storage, samples were periodically taken to measure the related physiological parameters.

### 2.2. Measurement of Physiological Parameters

Chlorophyll fluorescence was measured as previously described [45]. The protein carbonyl content was spectrophotometrically quantified using a carbonyl-specific reagent, 2, 4-dinitrophenylhydrazine. Malondialdehyde (MDA) content and total APX activity were determined using corresponding assay kits (Comin Biotechnology Co., Ltd., Suzhou, China) in accordance with the manufacturer’s instructions.

### 2.3. RNA Extraction, Gene Isolation, and Expression Analyses

Total RNA was extracted as previously described [46] and then subjected to reverse transcription-PCR. MaMsrB2 (GSMUA_Achr6T13640_001) and MaAPX1 (GSMUA_Achr5P07280_001) were isolated from a transcriptome database obtained using a SolexaHiSeqTM 2000 sequencing system. The gene-specific primers used for gene cloning are listed in Table S1. The PCR products were subcloned into a pMD20-T vector (TaKara) and then transformed into *E. coli* DH5α (TaKara) in accordance with the manufacturer’s protocol. The sequences were verified by further cloning and resequencing. Sequence alignments were carried out using ClustalX (version 1.83).

The qRT-PCR reactions for *MaMsrB2* and *MaAPX1* genes were carried out in the ABI 7500 Real-Time PCR System (Applied Biosystems, Carlsbad, CA, USA) with SYBR Green Real-Time PCR Master Mix (TOYOBO Co., Ltd.). The conditions were as followed: 30 s at 95 °C, 40 cycles of 5 s at 95 °C, and 34 s at 58 °C. *MaActin* was selected as the reference gene. qRT-PCR reactions were normalized using the Ct value corresponding to that of the reference gene. The relative expression levels of target genes were calculated using the formula 2−ΔΔCT. Three independent biological replicates were performed in the analysis. The primers used for gene expression analyses are listed in Table S1.

### 2.4. Generation of Transgenic Lines and Dark Treatment

The *MaAPX1* cDNA was sub-cloned in to a pCambia-1302 vector and then transformed into *E. coli* DH5α (TaKara) following the manufacturer’s protocol. The validated destination vectors were transformed into the *Agrobacterium tumefaciens* strain GV3101, and then transferred into the *apx1-2* Arabidopsis via the floral-dip method [47]. Homozygous transgenic progeny lines were obtained through hygromycin-resistance tests and RT-PCR analysis, and they were validated by analyzing the transcript levels of *MaAPX1-GFP* in the T3 generations. The gene-specific primers are listed in Table S1. Dark treatment of the detached leaves was performed as previously described [44]. Chlorophyll fluorescence was determined as per the above-mentioned method.

### 2.5. Subcellular Localization Analysis of MaMsrB2 and MaAPX1

The coding sequence fragments of *MaMsrB2* and *MaAPX1* without the stop codon were inserted into the pCambia1302 vector. Then, pCambia1302-*MaMsrB2*, pCambia1302-*MaAPX1,* or pCambia1302 (as the control) were transferred to Arabidopsis mesophyll protoplasts as previously described [48]. The green fluorescent protein (GFP) fluorescence was observed by a florescence microscope (Zeiss 510 Meta) after 18 h of incubation at 22 °C. All the transient expression assays were repeated at least three times.

### 2.6. Site-Directed Mutagenesis of Met36 Residues

The following site-directed mutagenesis in MaAPX1 were performed: Met36 to glutamine and valine using a PCR method [33]. The mutations were verified by DNA sequencing.

### 2.7. Preparation of Recombinant Proteins

The encoding sequence fragments of *MaMsrB2*, *MaAPX1,* or *MaAPX1* mutants were subcloned into the pGEX-4T-3 (Amersham Biosciences) or pET-28a vector (Novagen), respectively. MaMsrB2-GST, MaAPX1-His, MaAPX1-M36Q-His, and MaAPX1-M36V-His were induced and expressed in the *E. coli* BL21 (DE3) strain, and then were purified with glutathione sepharose 4B (GE Healthcare) and nickel-nitrilotriacetic acid agarose (Qiagen), respectively, following the manufacturer’s instructions (Figure S1).

### 2.8. Yeast Two-Hybrid (Y2H) Assay

The coding sequences of *MaAPX1* and *MaMsrB2* were subcloned into pGBKT7-BD or pGADT7-AD vectors to create bait and prey constructs. Then, the paired DNA-binding domain (BD) and transcription-activating domain (AD) constructs were co-transformed into the yeast strain AH109 (BioMed) using the lithium acetate method. Yeast cells were grown on minimal synthetic defined-double-dropouts (SD-Leu/-Trp) medium (Clontech) for 3 days. The co-transformants were then transferred onto quadruple dropout (QDO) (SD-Leu/-Trp/-Ade/-His) medium to test the possible interactions based on their growth status. The ability of yeast cells to grow on QDO medium was scored as a positive interaction.

### 2.9. Bimolecular Fluorescence Complementation (BiFC) Assay

The coding sequence fragments of *MaAPX1* and *MaMsrB2* without stop codons were inserted into pUC-pSPYCE or pUC-pSPYNE vectors. The fusion constructs were used for transient assays through a polyethylene glycol transfection of Arabidopsis mesophyll protoplasts, as described earlier [48]. The yellow fluorescent protein (YFP) fluorescence was observed by a fluorescence microscope (Zeiss 510 Meta) after 18 h of incubation at 22 °C.

### 2.10. GST Pull-down Assay

Equal volumes of GST-MaMsrB2 fusion protein or GST protein alone were incubated with the His-MaAPX1 fusion protein in pull-down buffer at 4 °C for 4 h, followed by incubation with GST beads for another 4 h. After washing with pull-down buffer three times, the pull-down proteins were boiled and subjected to SDS-PAGE and Western blot analysis using the anti-His antibody (TransGen Biotech). The signal was detected using a SuperSignal® West Pico Chemiluminescent Substrate (Thermo Fisher Scientific).

### 2.11. Oxidation and Reduction of MaAPX1

MaAPX1 protein (1 mg) was oxidized using H_2_O_2_ (10 mM) at 22 °C for 3 h in 50 mM PBS (pH 7.0). H_2_O_2_ was removed by centrifugal filter units (Amicon Ultra-10, Millipore). The repair of oxidized MaAPX1 was conducted by incubating oxidized proteins (2 μM of OX-MaAPX1) with MaMsrB2 (2 μM) and 10 mM DTT at 37 °C for 3 h. DTT was cleaned by centrifugal filter units. The proteins were collected and used for SDS-PAGE. Different redox statuses of MaAPX1 proteins were digested by trypsin, and then analyzed by LC-MS/MS on a C18 reverse-phase column. The redox status of each Met-containing peptide was analyzed using methods previously described by Jiang et al. [49].

### 2.12. Enzymatic Activity Measurement

Recombinant MaAPX1 activity was measured by spectrophotometry as previously described [50]. The activity was also evaluated by the nitroblue tetrazolium (NBT) method as previously described [51]. For activity analysis in vivo, the coding sequences of *MaAPX1*, *MaAPX1-M36Q,* and *MaAPX1-M36V* were cloned into the pCambia1302 vector. pCambia1302-*MaAPX1*, pCambia1302-*MaAPX1-M36Q*, pCambia1302-*MaAPX1-M36V*, or pCambia1302 (as the control) were transferred to Arabidopsis mesophyll protoplasts. After incubation for 18 h at 22 °C, the protoplasts were collected to analyze APX activity.

### 2.13. Spectroscopic Analysis

According to the method of Hugo et al. [52], UV-visible spectra were recorded at 25 °C from Asc-free MaAPX1, MaAPX1-M36Q, and MaAPX1-M36V (1 μg/μL) proteins in the presence or absence of an equimolar H_2_O_2_ concentration in potassium phosphate buffer (50 mM, pH 7.0).

### 2.14. Statistical Analysis

The data are presented as the mean ± SE of three biological replicates. Differences among different treatments were determined by ANOVA, followed by Dunnett’s test. Statistical analysis was performed using SPSS version 7.5 (SPSS, Inc., Chicago, IL, USA).

## 3. Results

### 3.1. Ripening Characteristics and Redox Status of Harvested Banana Fruit under High Oxygen Stress

**Figure 1:**
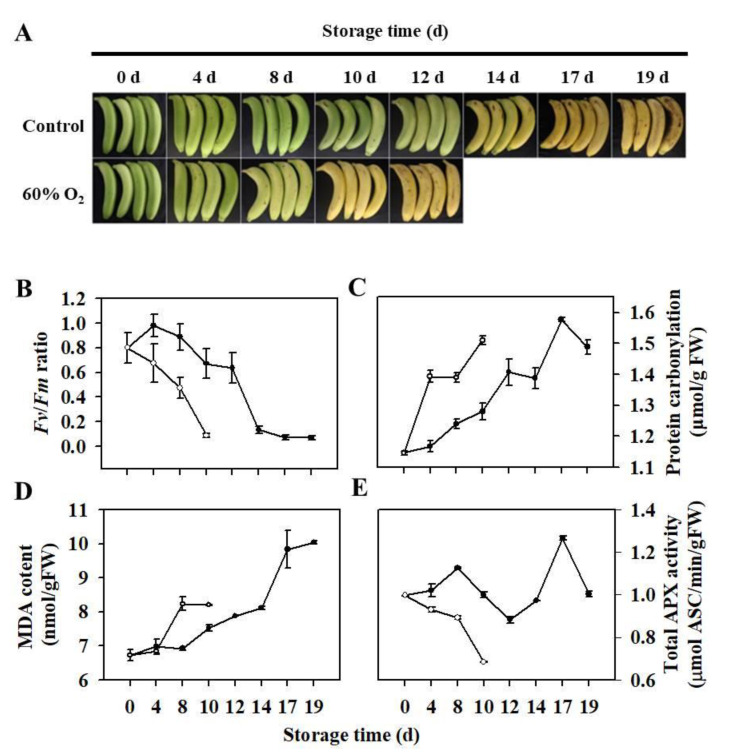
Ripening and senescence characteristics, and redox status of banana fruit stored under 60% O_2_ concentration. Fruit were stored under normal air conditions as the control. Changes in ripening phenotype (**A**), *Fv/Fm* (**B**), protein carbonylation (**C**), malondialdehyde (MDA) content (**D**), and ascorbate peroxidase (APX) activity (**E**) of banana fruit during storage.

Banana is a typical climacteric fruit characterized by a pre-climacteric phase followed by a peak in ethylene production that initiates ripening-associated processes. Of these processes, the peel turning yellow is one of the most important characteristics. As shown in Figure 1A, control fruit turned yellow at 14 d after harvest. The *Fv/Fm* indicates the efficiency in the energy transfer process and chloroplast activity, and it decreases in plants when subjected to abiotic stresses or during senescence. In this study, *Fv/Fm* in control fruit after 12 d of storage rapidly decreased (Figure 1B), implying thylakoid membrane damage, possibly due to senescence or oxidative stress. Protein carbonylation and MDA levels are important indexes of lipid and protein oxidation, respectively, which are usually used to evaluate oxidative stress in organisms. It was found that protein carbonylation level (Figure 1C) and MDA content (Figure 1D) in control fruit significantly increased with fruit ripening. Overall, harvested banana fruit ripening was accompanied by oxidative damage of proteins and lipids. 

We further mimicked oxidative stress by storing banana fruit at 60% oxygen concentration. High oxygen concentration accelerated ripening of harvested banana fruit, accompanied by more rapid decreases in *Fv/Fm* (Figure 1B). Compared with control fruit, fruit stored at 60% oxygen concentration exhibited more severe oxidative damage of proteins (Figure 1C) and lipids (Figure 1D). In addition, high oxygen concentration resulted in low APX activity in banana fruit (Figure 1E), which possibly results in a high accumulation of H_2_O_2_. It seemed that banana fruit ripening exhibited some oxidative stress-like attributes, implying that ROS played a role in banana fruit ripening. 

*MaMsrB2* and *MaAPX1* were isolated from a banana transcriptome database. Their transcript levels in the peel during fruit ripening and senescence were investigated by quantitative real-time PCR (qRT-PCR). The transcript level of *MaAPX1* initially increased and then decreased as ripening and senescence proceeded, whereas that of *MaMsrB2* tended to increase (Figure S2). High oxygen concentration accelerated the change in the transcript level of *MaAPX1* and *MaMsrB2*.

### 3.2. Overexpression of MaAPX1 in apx1-2 Delays Senescence under Dark Condition

**Figure 2:**
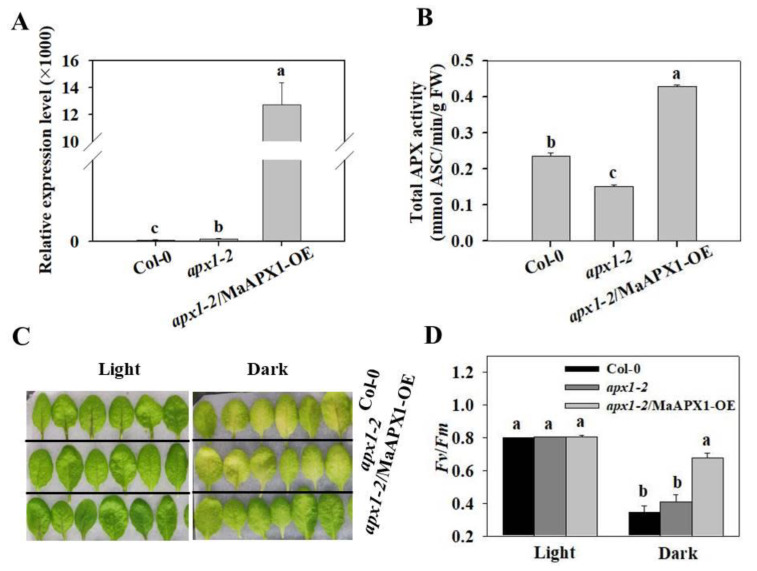
Ectopic overexpression of *MaAPX1* delays the detached leave senescence induced by darkness in Arabidopsis. (**A**) Expression of MaAPX1 in Col-0, *apx1-2,* and *apx1-2*/MaAPX1-OE seedlings. (**B**) Total APX activity in Col-0, *apx1-2,* and *apx1-2*/MaAPX1-OE seedlings. (**C**,**D**) Phenotype and *Fv/Fm* of detached leaves from three different genotypes incubated under dark or light (control) conditions for 5 d. Different letters above the bars indicate statistically significant differences between the samples (Student’s *t* test; *p* < 0.05).

The *APX* (GSMUA_Achr5P07280_001) cDNA, designated as *MaAPX1*, was isolated from banana fruit. *MaAPX1* was predicted to encode a 249-amino-acid protein with the characteristic sequence APLMLRLAWHSA in the active site, as well as binding sites of heme, substrate (AsA), and K^+^ (Figure S3). A previous study showed that APX1 in Arabidopsis is a central component of the reactive oxygen gene network, and the absence of APX1 led to an increase of H_2_O_2_ and an accumulation of oxidized proteins [53]. The *apx1-2* mutant showed various developmental defects, which are fully rescued by APX1 transgene complementary [50]. In the present study, we found that MaAPX1 had a high homology (83.9%) with Arabidopsis APX1. Homologs may have similar biological functions. We speculated that MaAPX1 may play a role in regulating senescence and oxidative stress. Therefore, we generated transgenic Arabidopsis plants overexpressing *MaAPX1* in the *apx1-2* mutant (Figure 2A). Enzyme activity analysis showed that the leaves of *apx1-2/*MaAPX1-OE had higher APX activity than those of *apx1-2* and Col-0 (Figure 2B). We further examined the senescence phenotypes of the detached leaf of Col-0, *apx1-2,* and *apx1-2/*MaAPX1-OE under dark conditions. As shown in Figure 2C, the leaves of the *apx1-2* and Col-0 were seriously yellow under dark conditions for 5 d, whereas only a slight yellow was observed in the *apx1-2/MaAPX1-OE*. Moreover, the *Fv/Fm* value of *apx1-2*/MaAPX1-OE was significantly higher than those of *apx1-2* and Col-0 (Figure 2D). These results indicated that ectopic overexpression of *MaAPX1* delays the detached leaf senescence induced by dark in Arabidopsis.

### 3.3. MaAPX1 Interacts Physically with MaMsrB2

**Figure 3:**
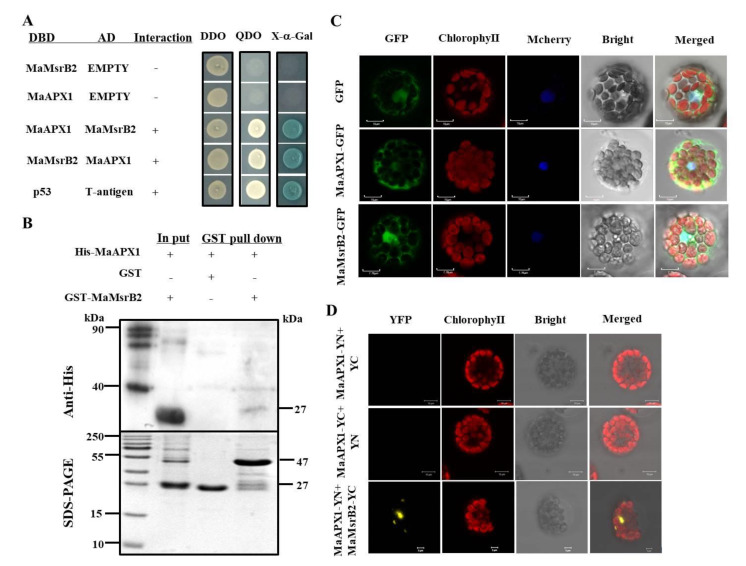
MaAPX1 interacts physically with MaMsrB2. (**A**) Interaction between MaAPX1 and MaMsrB2 in the yeast two-hybrid (Y2H) assay. (**B**) Interaction between MaAPX1 and MaMsrB2 in GST pull-down assay. The molecular weight of MaAPX1-His and MaMsrB2 is about 27-kDa and 47-kDa, respectively. (**C**) Subcellular localization of MaAPX1 and MaMsrB2. A green signal indicates green fluorescent protein (GFP) fluorescence; A red signal indicates chlorophyll auto fluorescence; The merged images represent a digital combination of chlorophyll auto fluorescence and GFP fluorescent images. (**D**) Interaction between MaAPX1 and MaMsrB2 in the bimolecular fluorescence complementation (BiFC) assay. A yellow signal indicates yellow fluorescent protein (YFP) fluorescence; a red signal indicates chlorophyll autofluorescence; the merged images represent a digital combination of the chlorophyll autofluorescence and YFP fluorescent images.

Three methods were applied to verify the interaction between MaAPX1 and MaMsrB2, including yeast two-hybrid (Y2H), bimolecular fluorescence complementation (BiFC), and pull-down assay. Y2H analysis showed that the yeast cells that were co-transformed with DNA-binding domain (DBD)-MaAPX1/activation domain (AD)-MaMsrB2 and DBD-MaMsrB2/AD-MaAPX1 grew well on minimal synthetic defined quadruple dropout (QDO) medium, while the DBD-MaAPX1/MaMsrB2 with AD did not grow (Figure 3A), indicating that MaAPX1 physically interact with MaMsrB2. Moreover, a pull-down assay showed that GST-MaMsrB2, but not GST alone, pulled down recombinant His-MaAPX1, indicating that MaAPX1 interacts with MaMsrB2 in vitro (Figure 3B). 

The subcellular localization analysis showed that MaAPX1 was mainly located in cytosol. In addition, MaAPX1 might also be distributed in the nucleus (Figure 3C). Furthermore, BiFC analysis further confirmed the interaction between MaAPX1 and MaMsrB2 in Arabidopsis mesophyll protoplasts. The interaction occurred in the nucleus and cytosol (Figure 3D), which was consistent with the subcellular localization of the two enzymes (Figure 3C).

### 3.4. MaMsrB2 Regulates Redox State and Activity of MaAPX1

**Figure 4:**
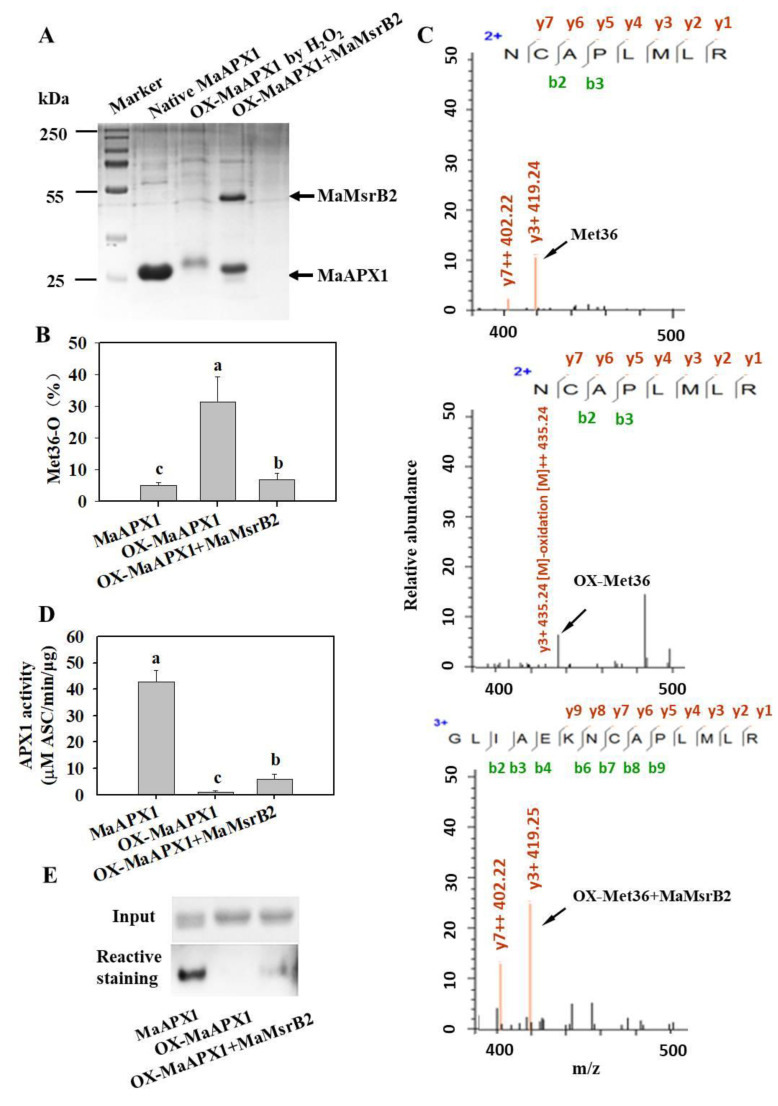
MaMsrB2 modifies the redox state and activity of MaAPX1. (**A**) Electrophoresis of native, oxidized, and reduced His-MaAPX1 in SDS-PAGE; (**B**) The percentage change of Met-O in the peptide containing Met36 with native, oxidized and reduced samples. Each bar represents the mean ± SE of three replicates. (**C**) Mass spectrometric analysis of the peptide containing Met36, Met36 oxidized by H_2_O_2_, and OX-Met36 reduced by MaMsrB2. (**D**,**E**) Activities of MaAPX1, oxidized MaAPX1, and MaMsrB2-repaired oxidized MaAPX1 by spectrophotometry (**D**) and reactive staining (**E**) methods. Different letters above the bars indicate statistically significant differences between the samples (Student’s *t* test; *P* < 0.05).

Recombinant MaAPX1 and MaMsrB2 proteins were prepared to determine whether MaMsrB2 can repair the oxidized MaAPX1. After oxidization with H_2_O_2_, the band of MaAPX1 in SDS-PAGE shifted to a higher molecular weight. When MaMsrB2 was added, the band of oxidized MaAPX1 almost moved back to the position of the native MaAPX1, suggesting that MaMsrB2 can reduce the oxidized MaAPX1 (Figure 4A).

Then, native MaAPX1, oxidized MaAPX1 and MaMsrB2-repaired oxidized MaAPX1 were submitted to trypsin digestion. LC-MS/MS analysis showed that the oxidized form of methionine significantly increased in the peptide containing Met36 in oxidized MaAPX1. When subjected to repair by MaMsrB2, the oxidized form of the peptide was reduced (Figure 4B). The detailed mass data of the peptide with different redox statuses are shown in Figure 4C. Collectively, these results indicated that oxidized MaAPX1 is a direct substrate of MaMsrB2 in vitro.

We further investigated whether Met oxidation in MaAPX1 affects the activity in vitro using the spectrophotometric method. As shown in Figure 4D, native MaAPX1 had an activity of 43.5 μM Asc mol^−1^ min^−1^ μg^−1^, whereas oxidation resulted in almost total loss of the MaAPX1 activity. When repaired by MaMsrB2, the activity restored 20% of the native MaAPX1. Moreover, similar results were also observed using the reactive staining method (Figure 4E). These results demonstrated that the repair of oxidized MaAPX1 by MaMsrB2 permits the recovery of its activity.

### 3.5. Mimicked Oxidation of Met in MaAPX1 Decreases the Activity of MaAPX1 In Vitro and In Vivo

**Figure 5:**
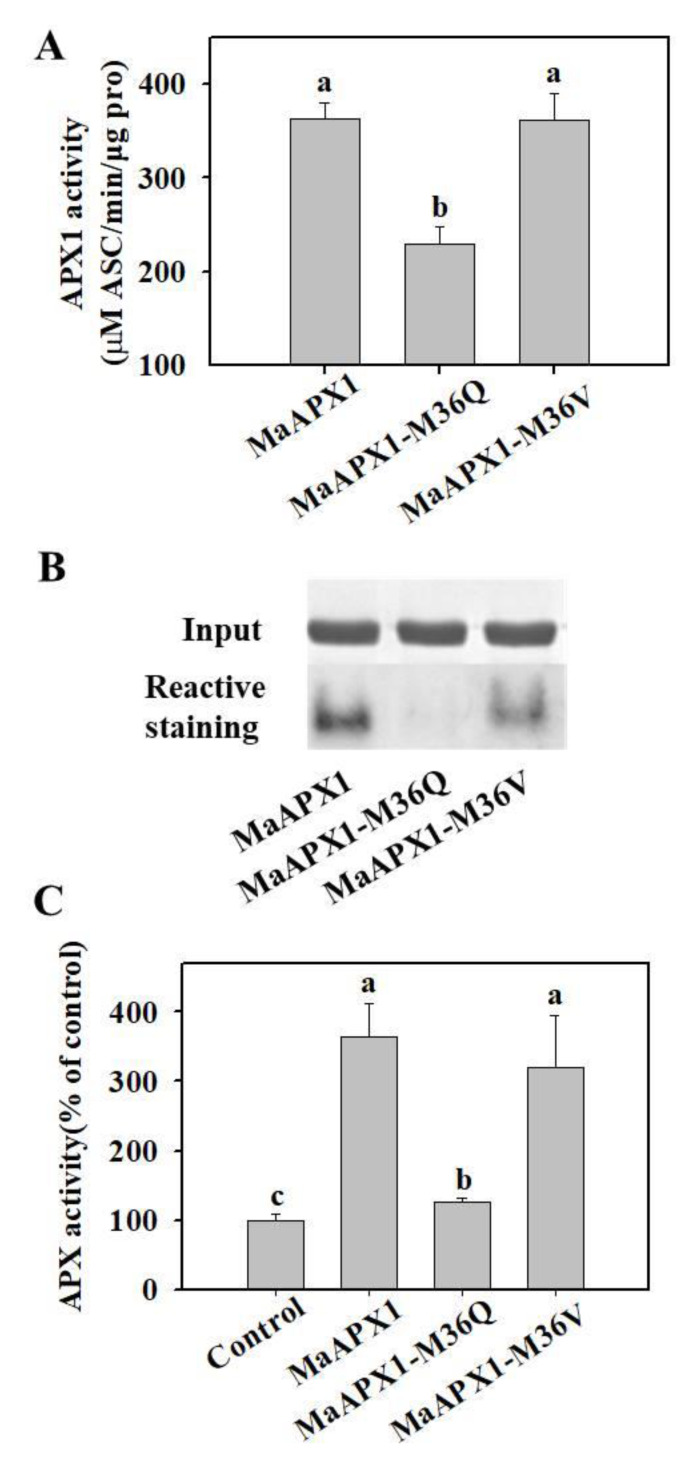
Mimicking sulfoxidation decreases MaAPX1 activity. MaAPX1-M36Q is a mutated form of MaAPX1 in which Met36 is mutated to glutamine to mimic sulfoxidation, whereas Met36 is mutated to valine in MaAPX1-M36V to mimic the blocking of sulfoxidation. (**A**,**B**) Activities of purified recombinant MaAPX1, MaAPX1-M36Q, and MaAPX1-M36V proteins by spectrophotometry (**A**) and reactive staining (**B**) methods. (**C**) The MaAPX1, MaAPX1-M36Q, and MaAPX1-M36V constructs was transformed into Arabidopsis mesophyll protoplasts. After incubation overnight at 22 °C, the protoplasts were collected to analyze APX activity by spectrophotometry. Different letters above the bars indicate statistically significant differences between the samples (Student’s *t* test; *p* < 0.05).

Met36 in MaAPX1 was highly conserved and located in the active site (Figure S3). We hypothesized that Met36 residue was an important site at which the redox status could influence the activity of MaAPX1. To confirm this hypothesis, we mutated Met36 residue to Gln36 to mimic methionine sulfoxidation, i.e., MaAPX1-M36Q, and mutated Met36 to Val36 to mimic the blocking of methionine sulfoxidation, i.e., MaAPX1-M36V. Recombinant MaAPX1, MaAPX1-M36Q, and MaAPX1-M36V proteins were prepared and their activities were analyzed. As shown in Figure 5A, mimicked oxidation of Met36 resulted in decreased APX activity, whereas there was no significant difference in APX activity between MaAPX1 and MaAPX1-M36V. The analysis by the reactive staining method also showed that mimicked oxidation of Met36 results in decreased APX activity (Figure 5B). 

Furthermore, MaAPX1, MaAPX1-M36Q, and MaAPX1-M36V proteins were expressed in Arabidopsis mesophyll protoplasts and their activities were analyzed. Compared with MaAPX1, mimicked oxidation of Met36 by mutant MaAPX1-M36Q significantly decreased APX activity, while mimicked blocking of Met36 sulfoxidation resulted in no significant influence on APX activity (Figure 5C).

### 3.6. Visible Spectral Analysis of MaAPX1, MaAPX1-M36Q, and MaAPX1-M36V Reacting with H_2_O_2_

**Figure 6:**
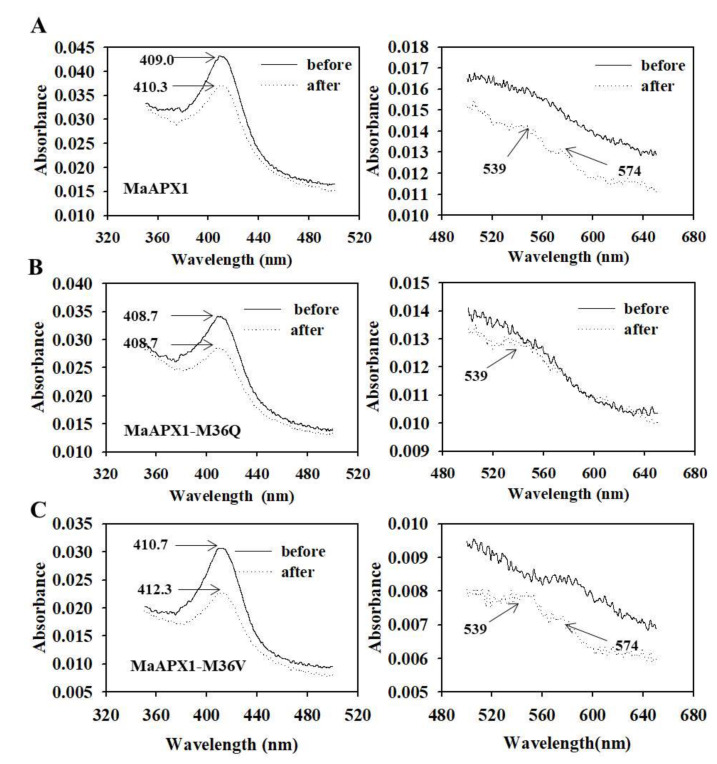
Absorption spectra of MaAPX1, MaAPX1-M36Q, and MaAPX1-M36V before and after H_2_O_2_ addition. (**A**) MaAPX1; (**B**) MaAPX1-M36Q; (**C**) MaAPX1-M36V. Left, spectra from 500 to 650 nm. Arrows indicate the Soret peak of the resting enzyme after the H_2_O_2_ reaction. Spectral shifts of the Soret peak are indicative of compound I-like formation. Right, spectra from 500 to 650 nm.

The optical spectra of MaAPX1, MaAPX1-M36Q, and MaAPX1-M36V (from 350 to 650 nm) were recorded before and after the addition of equimolar concentrations of H_2_O_2_. MaAPX1, MaAPX1-M36Q, and MaAPX1-M36V exhibited different Soret peaks at 409.0, 408.7, and 410.7 nm, respectively, indicating some structural perturbation at the heme microenvironment due to the amino acid replacement [52]. As shown in Figure 6, addition of H_2_O_2_ cause red shifts at the Soret region, which are associated with the formation of a compound I-like product [52,54,55]. The red shifts were observed in MaAPX1 and MaAPX1-M36V, accompanied by two humps at 539 and 574 nm. However, there was no shift at the Soret region for the mutant MaAPX1-M36Q, with only the 539 nm hump present (Figure 6).

## 4. Discussion

APX is a crucial antioxidant enzyme in regulating redox homeostasis. APX itself may also be subjected to multiple post-translational redox modifications, including S-nitrosylation, nitration, Cys oxidation, metal nitrosylation, glutathionylation, and carbonylation. Here, we characterized the role of MaAPX1 in regulating senescence by heterogeneous expression and elucidated a novel mechanism that is involved in post-translational redox modification of APX.

### 4.1. MaAPX 1 Might Be Involved in Ripening and Senescence in Relation to Oxidative Stress

Low oxygen concentration is the basis of modified-atmosphere or controlled-atmosphere storage for fruits and vegetables. High oxygen-modified atmospheres have been considered as an alternative preservation technique to classical low oxygen-modified atmosphere packaging. High O_2_ concentrations may retard, have no effect, or prompt fruit and vegetable ripening/senescence or deterioration, depending on the commodity, O_2_ concentration, and storage time [56,57,58,59,60,61,62,63]. In the present study, 60% O_2_ concentration resulted in lower APX activity, lipid peroxidation, and more severe protein oxidation, and accelerated fruit ripening in bananas. Qin et al. [43] reported that protein carbonylation increase in mitochondria during the senescence of peach fruit, and reducing ROS accumulation by low temperature inhibited protein carbonylation and retarded fruit senescence, whereas H_2_O_2_ treatment had the opposite effect. Li et al. [64] found that application of L-cysteine hydrochloride, a reactive oxygen species scavenger, suppressed aril breakdown and maintained fruit quality by reducing H_2_O_2_ accumulation and alleviating protein oxidation. Therefore, redox homeostasis is of importance for maintenance of fruit quality. Our results suggested that accelerated fruit ripening and senescence under high oxygen is associated with decreased APX activity, lipid peroxidation, and protein oxidation.

APXs are heme-containing peroxidases that catalyze reduction of H_2_O_2_ using ascorbate as the source of reducing power. APXs play key roles in regulating the precise localized concentration of H_2_O_2_ and are involved in physiological and developmental processes. Overexpression of APX in plants increases resistance to abiotic stresses, such as drought [65], salt [66], high temperature [67], and chilling injury [68]. Mutations of APX lead to increased sensitivity to oxidative stresses [69]. In the present study, genetic transformation of *apx1-2* mutant Arabidopsis with MaAPX1 obviously delayed the detached leave senescence induced by darkness, compared with the wild-type line. It is suggested that MaAPX1 plays a role in regulating senescence in plants.

### 4.2. MaAPX1 Is a Target of MaMsrB2

APXs in combination with other antioxidative enzymes and antioxidants regulate the homeostasis of ROS, especially H_2_O_2_. In plants, APXs are encoded by a multigene family. Arabidopsis, rice, and tomato contain nine, eight, and seven APX genes, respectively [70]. According to subcellular location, APX isozymes are classified into four categories, namely cytosolic, mitochondrial, chloroplastic, and peroxisomal APX [71]. In the present study, MaAPX1 was mainly localized in the cytoplasm and therefore belongs to cytosolic APX. Surprisingly, MaAPX1 was also detected in the nucleus. Under stress conditions or during aging, excessive accumulation of ROS leads to oxidative damage of proteins. Reversible oxidation of methionine in protein can be repaired by Msrs. In plants, MsrBs belong to one of two types, the 1-Cys type and the 2-Cys type based on the number of redox-active cysteines. *Arabidopsis thaliana* possesses nine *MsrB* genes and only AtMsrB1 belongs to the 1-Cys type [72]. MaMsrB2 had higher homology with AtMsrB1 than with other *A. thaliana* MsrBs (Figure S4). Consistently, MaMsrB2 has only one redox-active cysteine and is classified as the 1-Cys type (Figure S5). In *A. thaliana,* MsrBs are predicted to be located in cytosol and plastidial. Surprisingly, MaMsrB2 was distributed in cytosol and nucleus. The similar subcellular location enables the interaction between MaMsrB2 and MaAPX1. 

Msr-mediated repair of oxidized methionine in proteins is of importance for organisms in protecting against oxidative stress [34,38,39]. A great many proteins have been found through proteomics to be subjected to methionine oxidation [73,74]. However, only a few potential proteins have been validated as Msr targets in organisms [25,26,27,28,29,30,31,32,33,34,35,36,37]. Recently, Jiang et al. [49] found that E4/SlMsrB2 interacts with NON-RIPENING (NOR), an important ripening regulator, to regulate fruit ripening in tomato. In the present study, we verified using Y2H, BiFC, and pull-down assays that MaAPX1 interacted with MaMsrB2. Moreover, MaMsrB2 could reduce the oxidized methionine in MaAXP1. Interestingly, most of the MetO36 was reduced back to methionine. MaMsrB2 is expected to be stereospecific for the R-diastereomer. It seems that Met36 oxidation mainly resulted in the formation of R-diastereomer. These results indicated that MaAPX1 is a direct substrate of MaMsrB2 in bananas. Similarly, Tarrago et al. [75] used affinity chromatography to identify catalase, another enzyme that removes H_2_O_2_, as a potential target of AtMsrB1 in Arabidopsis.

### 4.3. MaMsrB2 Modifies the Redox Status of MaAPX1 and Affects Its Activity

Msr is implicated in sulfoxidation modification of proteins by reducing methionine sulfoxide to methionine. Previous studies on sulfoxidation modification have been mainly related to aging [76] and resistance to oxidative stresses [38,39]. Recently, it was considered that Msr-mediated redox modifications of proteins played a role as a post-translation modification [77]. The oxidation of methionine in proteins suppresses or improves their function, which can be reversed by Msr-mediated reduction [35,36,78]. More recently, Jiang et al. [49] reported that Met sulfoxidation in NOR, an important ripening-related transcriptional factor, or simulated sulfoxidation impairs its function in vitro, whereas E4 and SlMsrB2 partially reduce oxidized NOR and restore its function. Interestingly, methionine sulfoxidation in CaMKII activates its activity, whereas MsrA-mediated reduction of methionine sulfoxidation suppresses its activity in mice [29]. Similarly, simulated sulfoxidation of the hypochlorite-specific transcription factor HypT leads to the activated transcriptional activity in *E. coli* cells when subjected to HOCl stress, but the activity is suppressed by Msr-mediated reduction [31]. Therefore, Msr-mediated sulfoxidation modification in proteins is implicated in regulating their biological function.

APXs function as important regulators of redox balance in plant cells. However, APXs are susceptible to multiple redox-related post-translational modifications. Yang et al. [50] reported that S-nitrosylation of the Arabidopsis cytosolic ascorbate peroxidase1 (APX1) at cysteine (Cys)-32 enhances its activity of scavenging hydrogen peroxide, leading to the increased resistance to oxidative stress. Yamazaki et al. [79] identified cAPX as a potential target of Trx, and Gelhaye et al. [80] found that incubation of pea cAPX with reduced poplar Trxh, or reduced glutathione and dithiothreitol, significantly inactivate its activity, suggesting that activation of cAPX is related to Cys oxidation. Carbonylation of cAPX leads to an irreversible inhibition of APX activity [81]. Therefore, APX activity is regulated by multiple post-translation redox modification in plants. 

In this study, we found that methionine sulfoxidation in MaAPX1 inactivated its activity, which was reversed partially by MaMsrB2-mediated reduction of sulfoxidation (Figure 4D). Mimicking sulfoxidation of Met36, which is located in the active site, resulted in significantly decreased APX activity, whereas substitution of Met36 with Val36 to mimic the blocking of sulfoxidation had little effect on APX activity in vitro (Figure 5A,B). Similar results were also observed in Arabidopsis mesophyll protoplasts in vivo (Figure 5C). In APX-catalyzed reactions, APX first reacts with hydrogen peroxide to produce compound I, where the heme (iron V) is oxidized to the oxyferryl (Fe^4+^ = O) species, and then compound I returns to the resting ferric (Fe^3+^) state by two successive one-electron reactions with the substrate ascorbate [82]. Visible spectral analysis showed that mimicking sulfoxidation of Met36 hindered the formation of compound I, whereas substitution of Met36 with Val36 did not affect the formation of compound I. Our findings demonstrate that the redox state of methionine in MaAPX1 is of importance to its activity and MaMsrB2 can regulate the redox state and activity of MaAPX1 in bananas.

## 5. Conclusions

**Figure 7:**
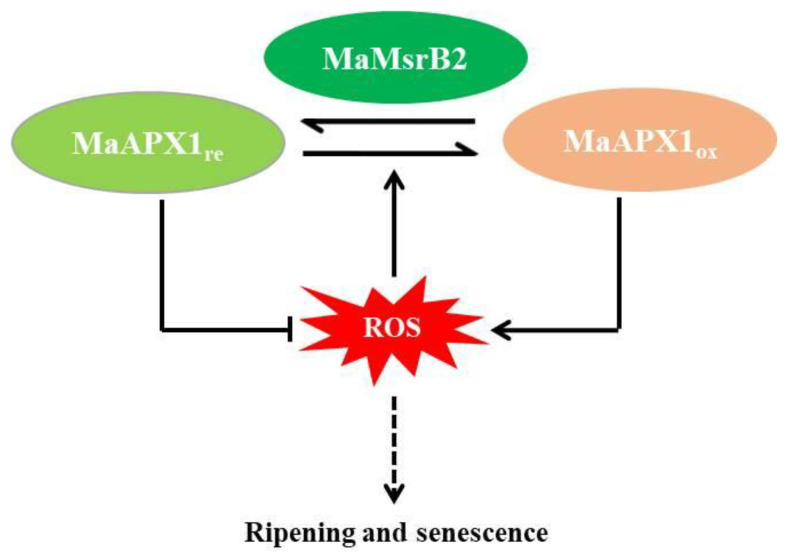
A proposed model of the involvement of MaMsrB2-mediated redox modification of methionine in MaAPX1 in regulating ripening and senescence.

In conclusion, we identified and confirmed that MaAPX1 is a substrate of MaMsrB2 in banana fruit. Ectopic overexpression of MaAPX1 delays the detached leaf senescence induced by darkness in Arabidopsis. Methionine sulfoxidation in MaAPX1 suppresses its activity, which can be partially restored by MaMsrB2. It is suggested that the MaMsrB2-mediated sulfoxidation modification of MaAPX1 possibly regulates redox status of banana fruit during ripening and senescence (Figure 7). Our results revealed a novel post-translational redox modification of APX.

## Data Availability

Data is contained within the article or supplementary material.

## Supplementary Materials

The supplementary are available online at https://www.mdpi.com/2076-3921/10/2/310/s1.
