# Supplementary material for: Methionine Sulfoxide Reductase B Regulates the Activity of Ascorbate Peroxidase of Banana Fruit
Source: Antioxidants (Basel). 2021 Feb 18;10(2):310. doi: 10.3390/antiox10020310 (PMC7922979; doi:10.3390/antiox10020310)
Supplement: Supplementary file 1 [file antioxidants-10-00310-s001.pdf]

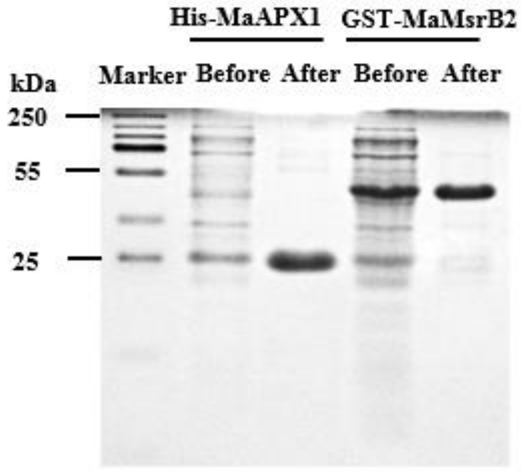

Figure S1 SDS-PAGE of expressed recombinant His-APX1 protein and GST-MaMsrB2 from *E. coli* BL21. “Before” and “After” referred to before and after protein purification.

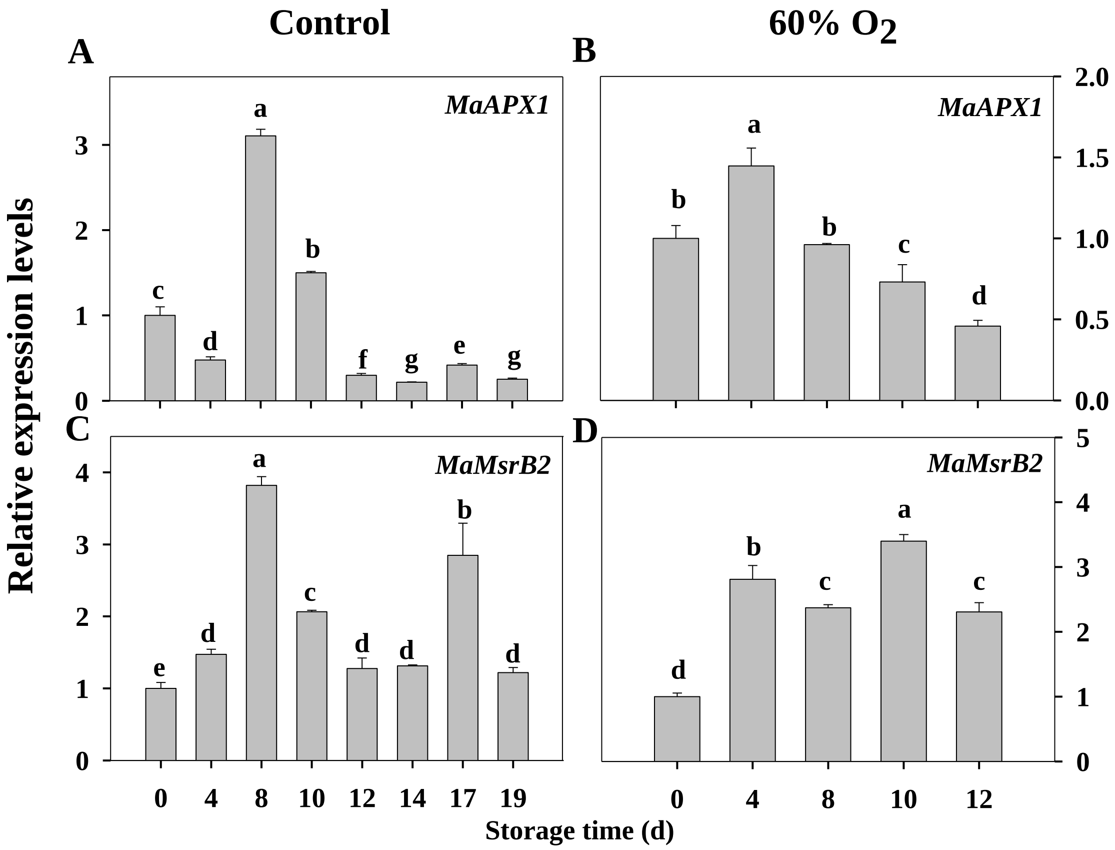

Figure S2 Expression of *MaAPX1* and *MaMsrB2* genes of banana fruit stored under air (control) or 60% oxygen at 25 °C. (A, C) control fruit. (B, D) fruit stored under 60% oxygen. The expression levels were expressed as a ratio relative to that of 0 d for control fruit, which was set as 1. Each value represents the mean ± SE of three biological replicates. Different letters indicated statistically significant differences between the samples (Student's t test; $P<0.05$).

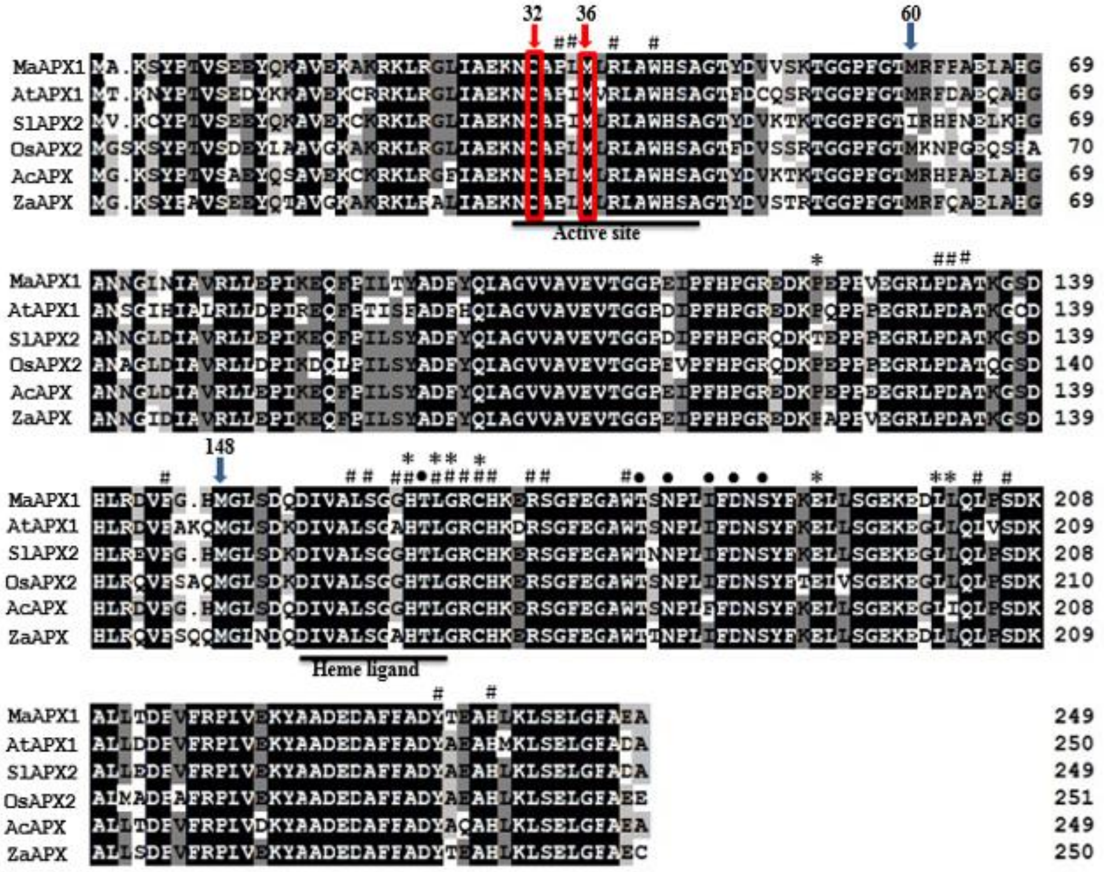

Figure S3 Multiple alignment of MaAPX1 with AtAPX1 (*Arabidopsis thaliana*, NP_001030991.2), SlAPX2 (*Solanum lycopersicum*, NP_001318094.1), OsAPX2 (*Oryza sativa*, XP_015646556.1), AcAPX (*Ananas comosus*, XP_020108209.1), ZaAPX (*Zantedeschia aethiopica*, AAC08576.1). #: heme binding site; *: substrate binding site; ●: K^+ binding site).

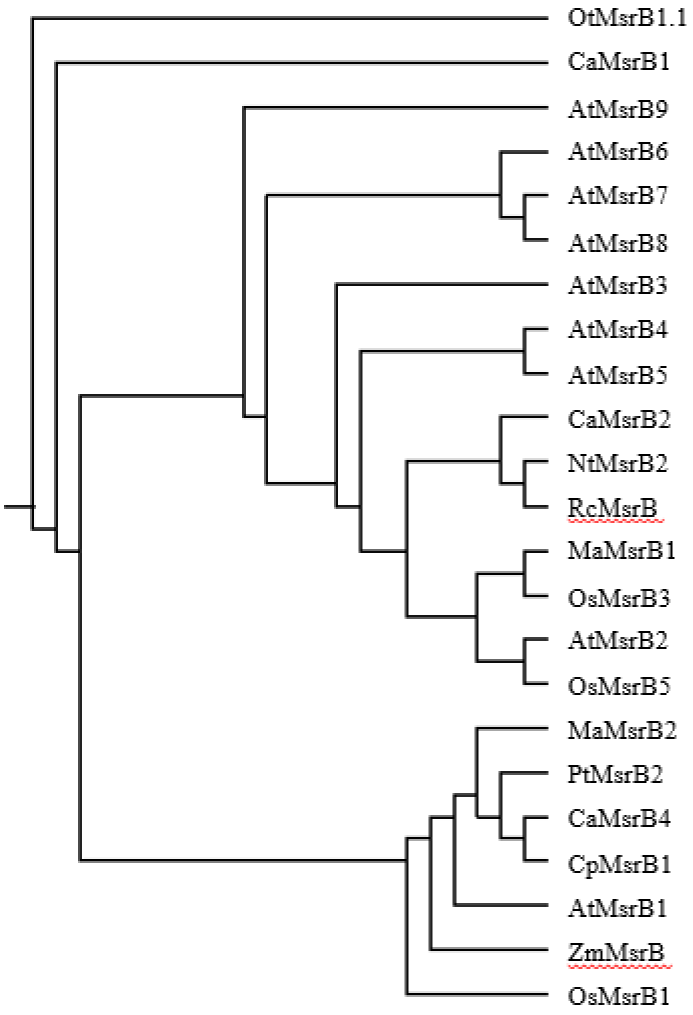

Figure S4 Phylogenetic alignment of MaMsrB1 and MaMsrB2 with MsrBs from *Arabidopsis thaliana*, *Capsicum annuum*, *Carica papaya*, *Medicago truncatula*, *Oropetium thomaeum*, *Oryza sativa*, *Populus trichocarpa*, *Ricinus communis* and *Zea mays*.

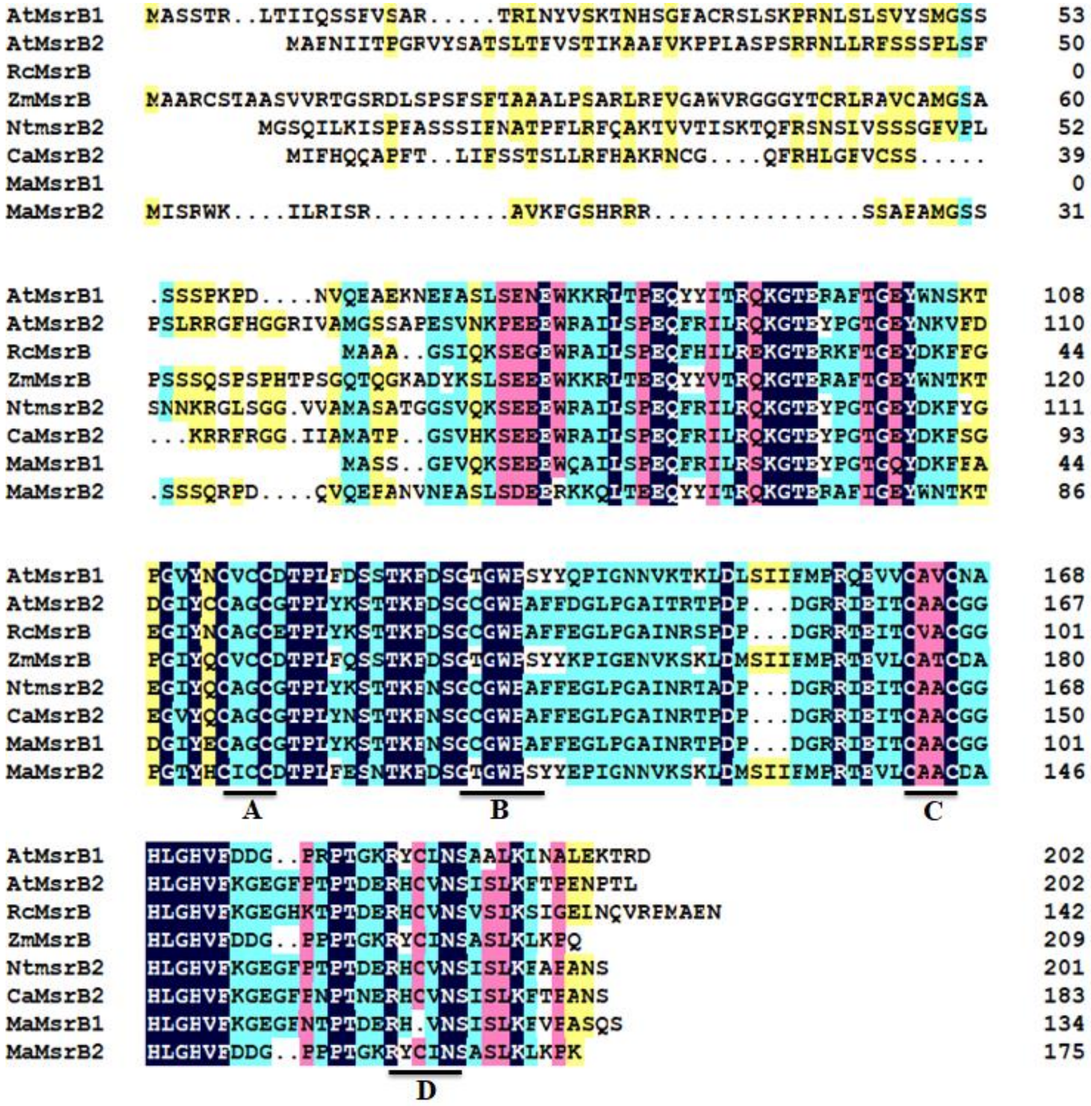

Figure S5 Multiple alignment of MaMsrB1 and MaMsrB2 with AtMsrB1, AtMsrB2, RcMsrB, ZmMsrB, NtMsrB2 and CaMsrB2. The conserved recycling motifs (B), conserved catalytic domain (D) and the zinc fixation motifs (A and C) are indicated by black lines.

Table S1 Summary of primers used in this study.

Gene	Forward(5'-3')	Reverse(5'-3')
**For full length cloning**		
MaAPX1	ATGGCGAAGTCGTATCCGACGG TGAGTG	TTAAGCCTCAGCAAATCCGAGTT CTGA
MaMsrB2	ATGATCAGTCGATGGAAAATCC TCCGC	CTACTTCGGCTTCAGCTTTAGGG ATGC
**For qRT-PCR in banana**		
MaAPX1	GGGCTCAACATCGCTGTCAGGC TCTT	GGCACCTTCCCAGTGTGTGTCCA CCA
MaMsrB2	TCTTTGTGCTGCCTGCGAT	CAGATGTCCGATAGATTTTGTC
MaActin	TGGTATGGAAGCCGCTGGTA	TCTGCTGGAATGTGCTGAGG
**For qRT-PCR in Arabidopsis**		
MaAPX1	TCTTGTGGAGAAATATGCTGCC	TTGGGACAACTCCAGTGAAA
AtUBQ	GATCTTTGCCGGAAAACAATTG GAGGATGG	CGACTTGTCATTAGAAAGAAAG AGATAACA
**For overexpression in Arabidopsis**		
MaAPX1-1302	GGACTCTTGACCATGGTAATGG CGAAGTCGTATCCGACGGTGA	GTCAGATCTACCATGGTAGCCTC AGCAAATCCGAGTTCT
**For subcellular localization**		
MaAPX1-GFP	GGACTCTTGACCATGGTAATGG CGAAGTCGTATCCGACGGTGA	GTCAGATCTACCATGGTAGCCTC AGCAAATCCGAGTTCT
MaMsrB2-GFP	GGACTCTTGACCATGGTAATGA TCAGTCGATGGAAAATCCTCCG C	GTCAGATCTACCATGGTCTACTT CGGCTTCAGCTTTAGGGATGC
**For site-directed mutagenesis of Met residues to glutamine and valine**		
MaAPX1M36Q	AACTGTGCCCCGTTGCAGCTCC GGCTC	GAGCCGGAGCTGCAACGGGGCA CAGTT

MaAPX1M36V	AACTGTGCCCCGTTGGTGCTCCGGCTC	GAGCCGGAGCACCAACGGGGCACAGTT
**For BiFC**		
MaAPX1-YC	CGCGCCACTAGTGGATCCATGGCGAAGTCGTATCCGACGGTGAGTG	GGGAGCGGTACCCTCGAGAGCCTCAGCAAATCCGAGTTCTGA
MaAPX1-YN	CGCGCCACTAGTGGATCCATGGCGAAGTGGACTTGAAGAAGTCGTG	GGGAGCGGTACCCTCGAGAGCCTCAGCAAATCCGAGTTCTGA
MaMsrB2-YC	CGCGCCACTAGTGGATCCATGATCAGTCGATGGAAAATCCTCCGC	GGGAGCGGTACCCTCGAGCTTCGGCTTCAGCTTTAGGGATGC
**For Y2H**		
MaAPX1-BD	GAGGAGGACCTGCATATGGCGAAGTCGTATCCGACGGTGAGTG	ACGGATCCCCGGGAATTCTTAAGCCTCAGCAAATCCGAGTTCT
MaAPX1-AD	CCAGATTACGCTCATATGGCGAAGTCGTATCCGACGGTGAGTG	CCCACCCGGGTGGAATTCTTAAGCCTCAGCAAATCCGAGTTCT
MaMsrB2-AD	CCAGATTACGCTCATATGATGATCAGTCGATGGAAAATCCTCCGC	CCCACCCGGGTGGAATTCCTACTTCGGCTTCAGCTTTAGGGATGC
MaMsrB2-BD	GAGGAGGACCTGCATATGATGATCAGTCGATGGAAAATCCTCCGC	ACGGATCCCCGGGAATTCCTACTTCGGCTTCAGCTTTAGGGATGC
**For protein expression**		
MaAPX1-His	CCGCGCGGCAGCCATATGGCGAAGTCGTATCCGACGGTGA	AGTCATGCTAGCCATATGTTAAGCCTCAGCAAATCCGAGTTCT
MaMsrB2-GST	CTGGTTCCGCGTGGATCCATGATCAGTCGATGGAAAATCCTCCGC	CCGGGAATTCGGGGATCCCTACTTCGGCTTCAGCTTTAGGGATGC
